# Intracellular Signaling Regulated by Activated α_2_-Macroglobulin: Expanding Beyond Its Protease Inhibitory Role

**DOI:** 10.3390/ijms27052487

**Published:** 2026-03-08

**Authors:** Lin Liu, Fang Yuan, Junting Jia, Yuyuan Ma

**Affiliations:** NMPA Key Laboratory for Quality Control of Blood Products, Academy of Military Medical Sciences, Beijing 100850, China; liulin5904@163.com (L.L.);

**Keywords:** alpha-2-macroglobulin, induced activation, LRP1, GRP78, intracellular signaling

## Abstract

Alpha-2-macroglobulin (α_2_M) is a conserved plasma glycoprotein traditionally known for its broad-spectrum protease inhibitory activity. However, emerging evidence indicates that its activated form, α_2_M*, generated via proteolytic cleavage or nucleophilic attack, functions as a versatile signaling ligand. By engaging specific cell-surface receptors, most notably low-density lipoprotein receptor-related protein 1 (LRP1) and glucose-regulated protein 78 (GRP78), α_2_M* orchestrates a diverse array of intracellular programs, including the PI3K/Akt/mTOR, MAPK/ERK, and JAK/STAT cascades, as well as mechanosensitive YAP/TAZ signaling. These pathways collectively govern fundamental cellular processes such as proliferation, metabolic reprogramming, cytoskeletal remodeling, and inflammatory adaptation across various cell types, including macrophages, cardiomyocytes, and malignant cells. Altogether, this review synthesizes current knowledge on α_2_M activation, structural transitions, receptor interactions, and downstream signaling, highlighting the expanding functional landscape of α_2_M* as a potent regulator of intracellular communication with implications for physiology and disease.

## 1. Introduction

Alpha-2-macroglobulin (α_2_M) is a large, multifunctional plasma glycoprotein [1], originally isolated in 1955 [2]. The native tetrameric molecule (~725 kDa) is composed of four identical subunits with complex domain organization, including macroglobulin-like domains (MG1–7), a bait region domain (BRD), a C1r/C1s-Uegf-Bmp1 (CUB) domain, a thioester domain (TED), and a receptor-binding domain (RBD) [3,4,5,6,7]. Post-translational modifications such as N-glycosylation contribute to structural stability and functional diversity [8]. α_2_M is widely expressed in hepatocytes, fibroblasts, astrocytes, and macrophages and is present in various body fluids [7,9].

Classically, α_2_M acts as a broad protease inhibitor, capable of trapping serine proteases, thiol proteases, and metalloproteases [10,11,12,13,14,15,16]. Beyond proteases, α_2_M binds cytokines [17], growth factors [18], and misfolded or aggregated proteins [19], functioning as a molecular carrier and extracellular chaperone [20]. These properties position α_2_M as a regulator of protein turnover, immune responses, and extracellular proteostasis.

In recent years, research has uncovered a much broader biological role for α_2_M, particularly in its activated form, α_2_M*. Upon proteolytic cleavage or nucleophilic attack, α_2_M undergoes dramatic conformational changes that expose its receptor-binding domain and enable signaling through cell-surface receptors. This review focuses on α_2_M activation mechanisms, the structural features of α_2_M*, and the signaling events it elicits through membrane receptors, aiming to delineate the emerging paradigm of α_2_M as a signaling mediator.

## 2. Induced Activation of α_2_M

### 2.1. Protease-Induced Transition to α_2_M*

**Figure 1:**
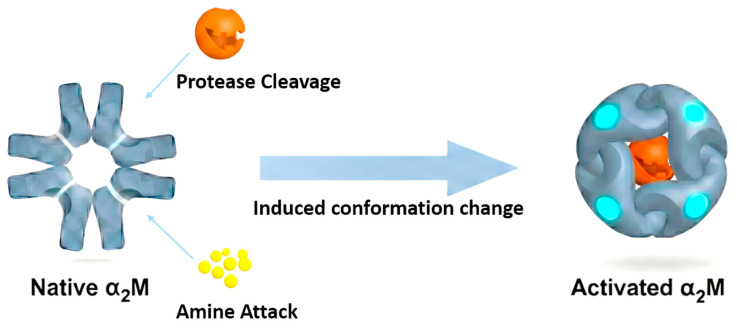
Induced activation of α_2_M. Native α_2_M undergoes conformational change upon induction by proteases or amines, which primarily act by cleaving the BRD (white segments in the native α_2_M tetramer). This transforms the protein from a loose tetrameric molecular cage into a tightly contracted encapsulating structure (α_2_M*), exposing the RBD (the cyan structural regions within α_2_M*) for subsequent membrane receptor engagement.

α_2_M inhibits proteases through a classical “Venus flytrap” mechanism: protease entry through the central cavity results in cleavage of the BRD, initiating a large-scale structural rearrangement that exposes the reactive thiol ester bond and drives coordinated rotations of the TED and CUB domains [21]. These conformational shifts progressively compact the molecule, creating a protease-entrapping cage (Figure 1). In some cases, nucleophilic residues of the protease can react with the exposed thioester, further stabilizing the α_2_M* state [22]. Cryo-electron microscopy (cryo-EM) analyses reveal substantial reorganization of inter-subunit contacts during this transition, providing structural insight into the enhanced receptor-binding capacity of α_2_M* [23].

### 2.2. Nucleophile-Induced Transition to α_2_M*

Small nucleophiles, such as methylamine (MA), can directly attack the thioester carbonyl to generate a non-protease-dependent activated form [24]. In these cases, though the bait region remains intact, the introduction of polar groups into the hydrophobic thioester site increases solvent accessibility [25], which facilitates a conformational collapse that closely resembles the one induced by protease cleavage and similarly exposes the RBD (Figure 1). Thus, despite the loss of its inhibitory capacity, this α_2_M* conformer retains the ability to act as a ligand for receptor-mediated signaling.

It should be noted that the majority of existing studies tend to treat both forms, that is, protease-α_2_M* and MA-α_2_M*, as functionally interchangeable activated conformations [21,24,26,27]. Nonetheless, the specific operational method of activation might introduce subtle yet critical heterogeneities in the resulting structures [28]. Specifically, protease-induced α_2_M* displays a distinct stain-excluding domain in its central part, indicating that the cavity is occupied by the protease; in contrast, MA-α_2_M* retains a longitudinal cavity, a structural divergence originally documented in the studies by Boisset et al. [29]. Meanwhile, the protease may partially protrude from the α_2_M* complex, resulting in a slightly larger Stokes radius compared to MA-α_2_M* [30]. Regarding reaction kinetics, the conformational transition triggered by the retraction of the N-terminal region of the truncated α-chain (α’NT) region from the central channel following bait-region cleavage occurs on a timescale of seconds, whereas the changes induced by aminolysis typically proceed over several minutes [31,32,33]. Protease-induced α_2_M* possesses greater energetic stability than MA-α_2_M*, as evidenced by Tm values of 68–69 °C versus 62.8 °C, respectively [34]. RBDs have been demonstrated to physically protrude from the molecular surface and exhibit significant dynamic flexibility in both conformers [21,35]; however, likely due to technical limitations, no studies have directly compared the potential differences in RBD exposure between these two activation forms. Thus, the α_2_M* label encompasses a spectrum of conformational states. In the following sections, we will specify the particular forms employed in each referenced study.

### 2.3. Hypochlorite Oxidative-Induced Transition to α_2_M Dimer

Hypochlorite, generated during inflammatory responses, induces oxidative modification of α_2_M that leads to tetramer dissociation into dimers with enhanced chaperone activity [36,37]. These oxidative-induced dimers exhibit a massive increase in surface hydrophobicity and increased affinity for cytokines and misfolded proteins, potentially exposing receptor-interacting surfaces independent of RBD-mediated activation [38,39]. Although these dimers do not undergo the canonical protease-triggered structural collapse and represent a structural departure from the classical tetrameric “fast” form, they constitute an alternative, functionally relevant species of α_2_M that operates predominantly under inflammatory or oxidative conditions.

### 2.4. Techniques for α_2_M* Characterization

A variety of biophysical and biochemical approaches have been used to distinguish α_2_M* from its native conformation. Early studies employing native-PAGE revealed that activation generates distinct, faster-migrating molecular species corresponding to α_2_M* [4,40]. The limitations of native-PAGE in distinguishing α_2_M species primarily stem from unresolved electrophoretic migration and non-specific mobility shifts. First, the presence of a single band on a gel does not necessarily guarantee a homogeneous population, as traditional fixed-percentage gels (e.g., 8% polyacrylamide) may lack the resolution to separate native and activated forms; in contrast, gradient gels (such as 3–8% or 4–12%) are often required to achieve adequate separation. Furthermore, mobility shifts on native-PAGE are not unique to a single activated state, as various structural alterations can result in similar migration patterns. Complementary spectroscopic analysis using circular dichroism (CD) further demonstrated that α_2_M activation is accompanied by characteristic alterations in secondary and tertiary structure, including changes in ellipticity and aromatic signals that reflect global domain rearrangements [24,41].

More detailed insights into conformational remodeling have been obtained through differential scanning calorimetry (DSC), which detects shifts in thermal stability between native, protease-activated, and MA-activated α_2_M*, reflecting structural rearrangements related to bait-region cleavage or covalent protease binding [34,42,43]. In addition, 4,4′-bis(1-anilinonaphthalene-8-sulfonate) (bis-ANS) fluorescence measurements reveal the exposure of hydrophobic surfaces upon activation or partial unfolding, allowing detection of subtle conformational transitions that may not be apparent in electrophoretic analyses [44,45]. However, the essence of this method remains an indicator of differences in surface hydrophobicity to infer the degree of conformational change; as such, it does not establish a one-to-one specific signature for predicting a particular activated form.

In sample preparation for various analytical methods, several experimental conditions, including temperature, buffer environment, gel concentration, protein concentration, detection time, and residual protease, can inadvertently trigger α_2_M conformational changes. For instance, the heat generated during prolonged high-current electrophoresis and the alkaline environment of loading buffers may induce minor α_2_M transformation [4,40]. Research has also shown that α_2_M undergoes slow inactivation when maintained in Tris-containing buffers for extended periods [40]; thus, detection times should be minimized as much as possible while still achieving experimental goals [24]. When utilizing CD, protein concentration should be optimized to balance signal intensity. Furthermore, Cummings et al. noted that residual protease within protease-α_2_M* complexes can alter the thermal transition properties of free α_2_M through its enzymatic activity, thereby confounding DSC results [34]. Consequently, purifying α_2_M* to remove impurities is essential for accurate peak assignment. Finally, as demonstrated by Wyatt et al., lyophilization or repeated freeze–thaw cycles significantly increase α_2_M hydrophobicity, indicating conformational alterations [45]. To mitigate this, samples should be aliquoted to avoid repeated freezing and thawing, or sucrose may be added as a cryoprotectant during lyophilized storage [45].

## 3. Receptor Interactions of α_2_M*

Activation of α_2_M exposes its C-terminal RBD, enabling interaction with specific cell-surface receptors that mediate both endocytic uptake and intracellular signaling. Among these receptors, LRP1 and GRP78 are the two best-characterized membrane receptors of α_2_M*, each contributing distinct regulatory functions. Their differential expression patterns and signaling capabilities allow α_2_M* to elicit diverse cellular responses across tissues.

### 3.1. LRP1

**Figure 2:**
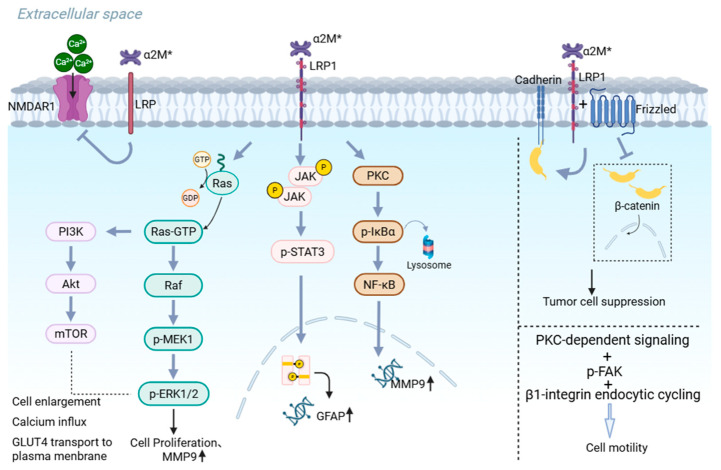
Schematic diagram of α_2_M* induced signaling via LRP1. Intracellular signaling pathways and associated biological effects triggered by the interaction between α_2_M* and LRP1. From left to right, the pathways are broadly categorized into phosphoinositide 3-kinase (PI3K) signaling, extracellular signal-regulated kinase 1/2 (ERK1/2) signaling, janus kinase-signal transducer and activator of transcription (JAK-STAT) 3 signaling, nuclear factor kappa B (NF-κB) signaling, Wnt-β-catenin signaling, and protein kinase C (PKC)-dependent signaling cascades, which collectively modulate various cellular processes including proliferation, survival, migration, and protein expression. Additionally, upon interaction with LRP1, α_2_M* can suppress NMDAR1 co-receptor-mediated calcium responses. Created with BioRender.com, with permission (https://BioRender.com/b3ahoqj (accessed on 2 February 2026)).

LRP1 is a multifunctional endocytic and signaling receptor that binds α_2_M* with high affinity. Mutational analyses have identified Lys-1370 and Lys-1374 within α_2_M* as essential residues for LRP1 recognition [46,47]. Following ligand binding, the α_2_M*-LRP1 complex undergoes rapid internalization, but ligand engagement also activates intracellular signaling pathways independently of endocytosis [48]. Importantly, LRP1 cooperates with co-receptors such as N-methyl-D-aspartate receptor (NMDAR) to generate ligand-specific responses, enabling α_2_M* to modulate processes ranging from inflammation to cell survival [49,50,51]. These properties position LRP1 as the principal mediator of α_2_M*-induced signaling in many cell types (Figure 2).

### 3.2. GRP78

**Figure 3:**
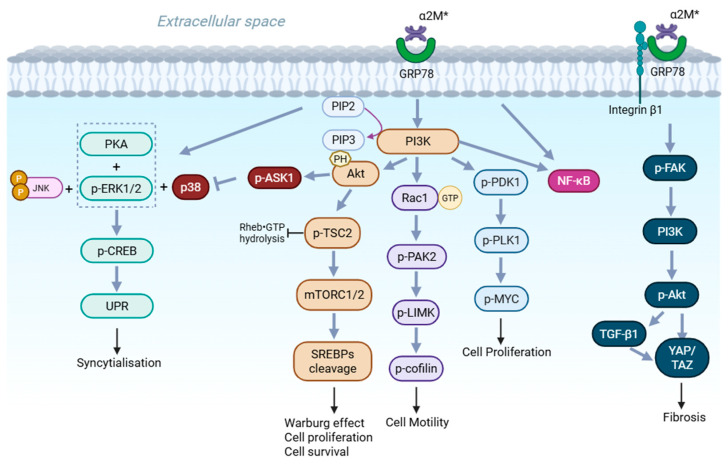
Schematic diagram of α_2_M* induced signaling via GRP78. Intracellular signaling pathways and associated biological effects triggered by the interaction between α_2_M* and GRP78. From left to right, the pathways are broadly categorized into mitogen-activated protein kinase (MAPK) signaling cascades (JNK, ERK1/2, p38), PI3K signaling cascades (Akt, PAK2, PDK1), and NF-κB signaling. In addition to their individual functional roles, cross-talks among these pathways collectively contribute to diverse cellular outcomes, including cell fusion, proliferation, survival, migration, and fibrosis. Created with BioRender.com, with permission (https://BioRender.com/b3ahoqj (accessed on 2 February 2026)).

GRP78, a member of the heat shock protein 70 family, is predominantly an endoplasmic reticulum (ER)-resident chaperone but can re-localize to the cell surface during stress [52]. At the plasma membrane, GRP78 functions as a high-sensitivity sensing system for extracellular stress signals and binds α_2_M* with sub-nanomolar affinity [53,54]. Mechanistically, the signal transduction capability of cell-surface GRP78 is anchored by its ability to organize specific signaling hubs within lipid raft microdomains. Specifically, in murine macrophages, α_2_M* ligation promotes the assembly of GRP78 into a ternary signaling complex with its transmembrane co-chaperone murine tumor cell DnaJ-like protein 1 (MTJ-1) and the Gαq11 subunit [55]. This complex formation is pertussis toxin-insensitive and indispensable for downstream signaling events, providing direct molecular evidence that GRP78 functions as a non-canonical G protein-coupled receptor (GPCR) adapter to transduce extracellular α_2_M* stimuli into intracellular responses [55]. Through this unique transduction mechanism, surface GRP78 modulates pathways governing survival, apoptosis, and inflammatory signaling, particularly in cancer cells and stressed tissues where its expression is significantly elevated [56]. Thus, GRP78 represents a context-dependent receptor that enables α_2_M* to orchestrate cellular adaptation within pathological signaling environments (Figure 3).

## 4. α_2_M*-Induced Intracellular Signaling

α_2_M* activates a diverse set of intracellular signaling pathways through interactions with LRP1, GRP78, and their associated co-receptors. Rather than functioning through a single linear pathway, α_2_M* engages interconnected signaling networks whose composition varies according to cellular context, receptor availability, and physiological state. Based on current evidence, these pathways can be organized into several functional modules: PI3K-driven growth and metabolic regulation, MAPK-family plasticity, Rac/PAK-dependent cytoskeletal remodeling, NF-κB-centered inflammatory control, JAK/STAT-mediated stress signaling, Wnt/*β*-catenin antagonism, and non-canonical YAP/TAZ mechanotransduction. This modular framework highlights the versatility of α_2_M* in coordinating cell-fate decisions across diverse tissues.

### 4.1. Growth- and Metabolism-Associated PI3K Modules

The PI3K-Akt axis serves as a robust central module for α_2_M* signaling, integrating extracellular stimuli into coordinated intracellular responses across diverse physiological contexts. Upon engaging cell-surface receptors, particularly GRP78, α_2_M* initiates a hierarchical signaling cascade that couples rapid kinase activation with long-term transcriptional reinforcement. For instance, the activation of Akt triggers the dual engagement of mTORC1 and mTORC2, which synergistically drive anabolic programs through S6K and 4EBP1 phosphorylation in 1-LN prostate cancer cells with 50 pM MA-α_2_M* [57]. This growth-promoting signaling is further amplified by a parallel PDK1-PLK1-MYC axis, where PDK1-mediated phosphorylation stabilizes the MYC oncoprotein to promote proliferative gene expression in various cancer cell lines (e.g., 1-LN and DU-145 prostate cancer, A375 melanoma, and U373 glioma cells) with 100 pM MA-α_2_M* [58]. The necessity of this receptor-specific pathway is underscored by the fact that GRP78 knockdown or PI3K inhibition effectively abolishes these downstream effects, confirming the module’s role as a primary driver of cellular expansion.

Beyond general growth control, the α_2_M*-driven PI3K-Akt axis acts as a master regulator of metabolic reprogramming. In malignant environments, specifically observed in 1-LN and DU-145 prostate cancer cells, treatment with 100 pM MA-α_2_M* induces a “Warburg-type” metabolic shift. This transition is characterized by enhanced aerobic glycolysis and a coordinated upregulation of lipogenic programs, including the SREBP1-c/SREBP2 axis and fatty acid synthase (FASN) [59]. This de novo lipid biosynthesis provides the necessary building blocks for rapid membrane biogenesis and tumor proliferation. Crucially, the oncogenic potential of this axis relies on the integrated activity of both mTOR complexes; pharmacological evidence using the dual inhibitor Torin1 demonstrates that simultaneous blockade of mTORC1 and mTORC2 is required to fully abrogate α_2_M*-mediated survival signaling, offering a more promising therapeutic strategy over traditional mTORC1-only inhibition [57].

The prominence of PI3K signaling is equally evident in tissue-specific responses to metabolic and hemodynamic stress. In the myocardium, ammonium bicarbonate-α_2_M* engages LRP1 to activate the PI3K-Akt-mTOR and ERK pathways, contributing to compensatory hypertrophy and metabolic adaptation [60,61]. This axis also fine-tunes systemic energy balance by modulating insulin sensitivity. 60 nM MA-α_2_M* facilitates glucose transporter type 4 (GLUT4) trafficking through Rab-dependent itineraries, thereby alleviating lipid-induced insulin resistance and enhancing glucose utilization efficiency in HL-1 cardiomyocytes [62,63]. However, under chronic pathological conditions such as diabetic kidney disease, this once-adaptive module can become maladaptive. In the renal microenvironment of type 1 diabetic Akita and CD1 mice, as well as human biopsy samples from diabetic kidney disease, high glucose levels have been shown to promote the formation of α_2_M*-GRP78 complexes [64]. Further mechanistic studies in mesangial cells demonstrate that 100 pM MA-α_2_M* sustains PI3K/Akt activation, which in turn drives the expression of profibrotic cytokines like transforming growth factor beta 1 (TGF-*β*1) and connective tissue growth factor (CTGF), ultimately leading to excessive extracellular matrix deposition and structural tissue pathology [64].

Also, the α_2_M*/LRP1-PI3K axis extends its regulatory reach to extracellular proteostasis and neuroprotection, particularly in the context of amyloid-beta (A*β*) metabolism. In amyloid precursor protein/presenilin 1 (APP/PS1) transgenic mice and neuroblastoma 2a cells, indomethacin promotes the expression of α_2_M. Upon activation by methylamine, 50 nM MA-α_2_M* triggers the PI3K-Akt and ERK1/2 pathways, which enhances a disintegrin and metalloproteinase 10 (ADAM10) activity for non-amyloidogenic APP processing and stabilizes LRP1 against A*β*-mediated degradation [65]. By facilitating the efflux and clearance of A*β* from the brain, this signaling module functions as a critical homeostatic mechanism against neurotoxic protein aggregation. Collectively, these findings position the PI3K-Akt axis as a recurring integration node through which α_2_M* coordinates cellular growth, metabolic flexibility, and the transition from homeostasis to disease.

### 4.2. MAPK-Family Regulatory Modules

MAPK signaling constitutes a second major node downstream of α_2_M*, functioning as a highly plastic regulatory hub that dictates cellular outcomes based on the specific receptor landscape and microenvironmental context. The functional versatility of this module is exemplified by its ability to oscillate between proliferative and anti-inflammatory roles. In mitogenic contexts, 60 nM MA-α_2_M* triggers ERK1/2 and c-jun activation primarily through LRP1 to drive cell cycle progression in J774 cells [66,67]. However, the assembly of a distinct LRP1-NMDAR signaling complex can pivot this response toward immune modulation, where ERK1/2 activation serves to suppress the production of pro-inflammatory cytokines [51]. This flexibility underscores the “context-dependent” nature of α_2_M* activity, where the co-receptor composition determines whether the MAPK cascade promotes expansion or resolution.

The regulatory reach of α_2_M* is further amplified through the extensive integration of MAPK modules with lipid mediators and cyclic nucleotide signaling. Within the immune microenvironment, 100 pM MA-α_2_M* triggers a phospholipase C (PLC)-dependent mobilization of intracellular Ca^2+^, which sequentially activates protein kinase C (PKC) and the broader MAPK family, including ERK, p38, and JNK. This network converges on the phosphorylation and translocation of cytosolic phospholipase A2 (cPLA_2_), thereby linking kinase cascades to arachidonic acid release and the transcriptional induction of *c-fos* and *c-myc* in mouse peritoneal macrophages [68]. Concurrently, α_2_M* orchestrates a multi-axis signaling program that integrates Ca^2+^-dependent pathways with cAMP signaling. Specifically, in macrophages, treatment with 100 pM MA-α_2_M* triggers the parallel activation of the PKA, PI3K, and MAPK pathways. This concerted signaling network converges on the phosphorylation of cAMP responsive element binding protein (CREB), establishing it as a central node for driving immune cell proliferation and survival [69].

Beyond basic growth control, this signaling architecture is essential for specialized cellular differentiation and developmental programs. A striking example is found in placental development, where α_2_M* engages a specific GRP78-ERK/CREB axis that uniquely couples with the unfolded protein response (UPR). This mechanistic integration, independent of canonical human chorionic gonadotropin (hCG) secretion, is indispensable for regulating trophoblast fusion, providing a novel paradigm for how 100 pM trypsin-α_2_M* or MA-α_2_M* coordinates extracellular stimuli with ER-stress pathways to govern tissue morphogenesis [70]. Such layered regulation is also evident in malignant and inflammatory states, where ERK activation frequently coincides with the recruitment of Akt, p38, and NF-κB triggered by picomolar to nanomolar concentrations of MA-α_2_M*, forming a robust survival network that reinforces tumor resistance and matrix remodeling through the induction of matrix metalloproteinase-9 (MMP-9) [71,72,73].

The α_2_M*-MAPK module functions as a critical homeostatic rheostat in neural and ocular tissues. In retinal environments, 100 nM MA-α_2_M* acts as a suppressor of uncontrolled glial proliferation by antagonizing GPCR-induced mitogenic signaling. This is achieved through a dual mechanism: the LRP1-mediated endocytic clearance of extracellular growth factors and proteases, coupled with the attenuation of Ca^2+^-dependent signaling and the stabilization of membrane conductance in Müller cells, thereby preventing the overactivation of downstream MAPK-dependent proliferative pathways [74]. This homeostatic counter-regulation highlights the importance of α_2_M* in maintaining retinal stability and preventing pathological states such as proliferative vitreoretinopathy. Collectively, these findings illustrate that the MAPK module is not a linear pathway but a multi-dimensional integration point that fine-tunes cell fate, differentiation, and tissue homeostasis.

### 4.3. Rac-PAK-Dependent Cytoskeletal Remodeling Modules

Beyond its roles in growth and metabolism, α_2_M* serves as a key regulator of cytoskeletal architecture, acting largely through a Rac1-centered signaling axis. The fundamental framework of this module involves the activation of Rac1 upon 50 pM MA-α_2_M* binding to surface GRP78, which subsequently recruits p21-activated kinase 2 (PAK2). This interaction releases the autoinhibitory conformation of PAK2, enabling its autophosphorylation (Thr-402) and the subsequent activation of the LIM domain kinase (LIMK)-cofilin cascade in murine peritoneal macrophages [75]. By inhibiting the actin-filament-severing activity of cofilin, this pathway stabilizes F-actin structures, providing the mechanical basis for cellular protrusions and directed migration.

Recent mechanistic dissections have refined this model by identifying a high degree of isoform specificity and the requirement for specialized adapter proteins. In the context of malignant progression, 50–100 pM MA-α_2_M* drives the assembly of a plasma membrane-associated ternary complex consisting of GRP78, the adapter protein non-catalytic region of tyrosine kinase adaptor protein (NCK), and PAK2 in 1-LN cells [76]. Notably, this signaling requirement is specific to PAK2, as PAK1 cannot substitute in this context. This specialized GRP78-NCK-PAK2 module serves as a dual-function hub: while it regulates actin dynamics through the LIMK-cofilin axis to facilitate invasion, it simultaneously phosphorylates the pro-apoptotic protein Bad to suppress programmed cell death. This sophisticated coupling of cytoskeletal reorganization with survival signaling suggests that α_2_M* does not merely move a cell but actively sustains its viability during the high-stress process of metastasis.

The versatility of α_2_M*-driven motility is further exemplified by its ability to coordinate complex mesenchymal migration programs. This is particularly evident during tissue remodeling and inflammatory infiltration, where 60 nM MA-α_2_M* engagement of LRP1 activates a PKC-dependent motility program in Raw264.7 cells [77]. Unlike simple chemotaxis, this axis involves the active redistribution of membrane-type 1 matrix metalloproteinase (MT1-MMP) and the rapid endocytic cycling of *β*1-integrin to the leading edge of cellular protrusions. By integrating proteolytic activity (via MT1-MMP) with adhesive dynamics (via integrins) and mechanical force, the α_2_M*/LRP1 pathway enables cells to navigate through dense extracellular matrices. Collectively, these findings position Rac-PAK-dependent modules as critical integration points where α_2_M* facilitates the transition from stationary to invasive cellular phenotypes in both physiological and pathological environments.

### 4.4. NF-κB-Driven Inflammatory Modules

The NF-κB transcription factor family represents a critical convergence hub through which α_2_M* translates acute extracellular stimuli into sustained changes in the cellular transcriptional landscape. In macrophages, 20 nM MA-α_2_M* initiates a PKC-dependent cascade that drives the nuclear translocation of NF-κB, leading to the targeted induction of MMP-9 [71]. Rather than acting in isolation, this pathway is closely integrated with the ERK1/2 signaling module, forming a coordinated regulatory program that governs macrophage-mediated matrix remodeling and tissue infiltration.

The functional significance of the NF-κB module is further amplified in malignant settings, where it operates as a cornerstone of cellular resilience. In this context, 50 pM MA-α_2_M* generates a high-order, multi-axis signaling network by simultaneously engaging NF-κB alongside the PI3K/Akt, ERK1/2, and p38 MAPK pathways [72,73]. This integrative signaling program may confer a notable survival advantage, helping cancer cells to maintain proliferative momentum and counteract environmental stressors or apoptotic triggers. By positioning NF-κB at the intersection of growth, survival, and stress-response signaling, α_2_M* appears to function as a broad-spectrum modulator of gene expression, potentially bridging the gap between mitogenic signaling and inflammatory adaptation.

### 4.5. JAK-STAT-Mediated Stress-Response Modules

α_2_M* also modulates cellular responses to stress via activation of JAK-STAT signaling. In retinal Müller glial cells, 60 nM MA-α_2_M* binding to LRP1 triggers time-dependent STAT3 phosphorylation, leading to increased expression of glial fibrillary acidic protein (GFAP)—an established indicator of glial reactivity. This pathway’s physiological relevance is validated in vivo, where intravitreal 1.075 μg per mouse MA-α_2_M* recapitulates STAT3-dependent GFAP induction, identifying α_2_M* as a key molecular sentinel in neural tissues [78,79]. JAK-STAT signaling represents a conserved stress-response mechanism that may extend to other tissues. By modulating STAT3 activity, α_2_M* may influence broader aspects of tissue homeostasis.

### 4.6. Wnt/β-Catenin Antagonism Modules

In certain pathological contexts, such as glial malignancies, 0–0.1 μM MA-α_2_M* can function as a negative regulator of the canonical Wnt/*β*-catenin pathway. This inhibitory effect is mediated through the engagement of LRP1, which triggers the sequestration of Frizzled receptors and a concomitant up-regulation of E-cadherin and N-cadherin [71]. These molecular changes promote the redistribution of *β*-catenin, shifting its localization from the nucleus to the plasma membrane. By reducing the nuclear pool of *β*-catenin, this module effectively suppresses Wnt-driven transcriptional programs [80], thereby limiting cellular invasive potential and the formation of multicellular spheroids. Such findings suggest that the α_2_M*/LRP1 axis may act as a biological constraint on tumor progression, particularly within the nervous system [81].

### 4.7. Non-Canonical Profibrotic Signaling

Beyond classical kinase cascades, α_2_M* participates in the regulation of tissue fibrosis through non-canonical signaling pathways. In renal tubular epithelial cells and fibroblasts, the interaction between protease-α_2_M* and cell-surface GRP78 is a required step for profibrotic responses induced by high glucose levels. Although this axis intersects with TGF-β signaling, its downstream effects are independent of the canonical Smad3 pathway [82]. Instead, protease-α_2_M* signaling through GRP78 promotes the activation of the YAP-TAZ transcriptional complex, a key mediator of fibrosis and extracellular matrix deposition [82,83]. In vivo studies have demonstrated that pharmacological or genetic blockade of the protease-α_2_M*/GRP78 interaction effectively suppresses this pathway and attenuates the progression of renal fibrosis. These findings identify α_2_M* as a modulator of matrix-associated transcriptional programs, potentially linking extracellular ligand status to mechanosensitive signaling components like YAP/TAZ [82].

## 5. Perspective

α_2_M has long been viewed primarily as a broad-spectrum protease inhibitor, yet recent structural and mechanistic advances reveal that its activated form, α_2_M*, plays a far more expansive biological role. The signaling landscape of α_2_M* appears to be characterized by a remarkable pleiotropy, often engaging multiple intracellular cascades at concentrations as low as the picomolar to nanomolar range. In certain cell types, these α_2_M*-mediated responses may exhibit distinct dose-dependent activation patterns, suggesting a potentially sensitive and finely regulated mechanism for modulating extracellular signals. As summarized in Table 1, α_2_M* modulates a wide array of pathways, including the classical kinase cascades (MAPK/ERK, PI3K/Akt), master transcriptional regulators (NF-κB, STAT3, Wnt/β-catenin), mechanosensitive modules (YAP/TAZ), and neuronal function [84]. This functional diversity is largely dictated by the specific receptor-ligand interface and the cellular context. Evidence suggests that LRP1 remains a predominant and highly versatile receptor for α_2_M*, functioning as a signaling scaffold in macrophages, glial cells, and various tumor lineages. Within these cells, it can trigger divergent downstream effects depending on the physiological state. In contrast, the α_2_M*/GRP78 axis appears to represent a more specialized signaling module, predominantly observed in epithelial cells and fibroblasts.

Despite substantial progress, several major gaps continue to constrain our understanding of α_2_M* biology. A primary challenge in α_2_M* biology is deciphering the molecular logic that governs signaling specificity. That is, α_2_M* can elicit starkly divergent outcomes (i.e., enhancing survival in cancer cells, suppressing inflammation in macrophages, or supporting homeostatic responses in neuronal cells), yet the molecular logic underlying these differences remains unknown. Also, it remains unclear how different conformational states alter receptor engagement and pathway choice. Establishing a rigorous structural framework that links specific conformational ensembles to receptor or pathway selection will be attractive for the field.

Another intriguing aspect to note lies in the clinical relevance of α_2_M* dysregulation. Altered α_2_M levels have been associated with neurodegeneration, bladder cancer, and bone disorders [20,85,86,87], yet the biological significance of these correlations remains opaque. It is not known whether such changes reflect compensatory responses to systemic stress, contribute causally to disease progression, or simply act as biomarkers of upstream dysfunction. The extent to which α_2_M* contributes to disease heterogeneity also remains unexplored. Emerging methodologies offer the precision required to map α_2_M* signaling dynamics in complex tissue environments. Specifically, high-resolution cryo-EM could be employed to elucidate the structural divergence between α_2_M activated via different pathways, specifically focusing on the exposure of the RBD and how these conformational nuances dictate its interaction with receptors. Live-cell biosensors could test hypotheses regarding the spatiotemporal activation of α_2_M within the microenvironment of living tissues. These tools will be instrumental in validating α_2_M* as a viable therapeutic target. As α_2_M* continues to emerge as a versatile regulator of extracellular signaling, defining its mechanistic diversity will be essential for translating fundamental discoveries into clinical impact.

## Figures and Tables

**Table 1:** Summary of signaling and biological effects induced by α_2_M* interaction in different cells.

Experimental System	Cell Line	Stimulator	Dosage	Induced Signaling	Biological Effects	Mediating Receptor	Receptor Validation	Reference
In vitro (murine prostate cancer cells)	1-LN	MA-α_2_M*	50 pM	PI3K activation, Akt1 phosphorylation, mTORC1/2 activation, S6K/4EBP1 phosphorylation	Cell proliferation	GRP78	α-GRP78	[57]
In vitro (murine and human prostate cancer cells, human melanoma cells, human glioma cells )	1-LN, DU145, A375, U373	MA-α_2_M*	100 pM	PDK1 phosphorylation, PLK1 phosphorylation, c-MYC phosphorylation, target genes transcription	Cell proliferation, Cell survival	GRP78	α-GRP78, GRP78 mutation	[58]
In vitro (rat ventricular cardiomyocytes)	Primary cells	Ammonium bicarbonate-α_2_M*	/	ERK1/2 activation, PI3Kactivation, Akt activation	Hypertrophic cell growth, Contractile responsiveness	LRP1	RAP	[61]
In vitro (murine cardiomyocytes)	HL-1	MA-α_2_M*	60 nM	ERK phosphorylation, Akt phosphorylation, Rab4/Rab8A/Rab10 GTPase activation	Improved insulin response	LRP1	α-LRP1	[63]
In vitro (murine and human prostate cancer cells)	1-LN, DU145	MA-α_2_M*	100 pM	PI3K activation, Akt activation, mTORC activation, SREBPs cleavage, FASN	Cell proliferation, Warburg effect	GRP78	α-GRP78, dsRNA-mediated GRP78 knockdown	[59]
In vitro (murine mesangial cells, T2D-DKD urine); in vivo (type 1 diabetic Akita and CD1 mice)	Primary cells	MA-α_2_M*	100 pM	Akt phosphorylation, TGF-β1 activation, ECM protein upregulation, CTGF expression	Fibrogenesis	GRP78	Peptide-mediated GRP78 blockade	[64]
In vitro (murine macrophage cells)	J774	MA-α_2_M*	60 nM	ERK1/2 phosphorylation, c-jun phosphorylation	Cell proliferation	LRP1	RAP, reducing LRP1 expression using LPS	[66]
In vitro (murine macrophage cells)	J774, Raw264.7	MA-α_2_M*	20 nM	PKC activation, ERK1/2 activation, NF-κB activation	MMP-9 expression	LRP1	RAP	[71]
In vitro (murine prostate cancer cells)	1-LN	MA-α_2_M*	50 pM	Akt phosphorylation, ERK1/2 phosphorylation, p38 MAPK activation, NF-κB activation	Cell proliferation, Anti-apoptotic	GRP78	α-GRP78, dsRNA-mediated GRP78 knockdown	[72]
In vitro (human choriocarcinoma cells)	BeWo	MA-α_2_M*, trypsin-α_2_M*	100 pM	ERK1/2 phosphorylation, PKA activation, CREB phosphorylation, UPR activation, JNK phosphorylation	Syncytialization	GRP78	α-GRP78	[70]
In vitro (murine bone marrow-derived macrophages)	Primary cells	MA-α_2_M*	2–120 nM	Reduced IκBα phosphorylation, Src family kinases activation, ERK1/2 phosphorylation	Antagonism of LPS-induced response	LRP1	LRP1 knockout	[51]
In vitro (human Müller cells); in vivo (C57BL/6 mice)	MIO-M1	MA-α_2_M*	60 nM, 1.075 μg per mouse	STAT3 phosphorylation	GFAP expression	LRP1	α-LRP1, RAP	[78]
In vitro (murine peritoneal macrophages)	Primary cells	MA-α_2_M*	50 pM	RasGAP upregulation, Rac-1 activation, NCK recruitment, PAK2 phosphorylation, LIMK phosphorylation, cofilin phosphorylation, PI3K phosphorylation	Cellular motility	GRP78	α-GRP78, dsRNA-mediated GRP78 knockdown	[75]
In vitro (murine prostate cancer cells)	1-LN	MA-α_2_M*	50–100 pM	PI3K activation, PAK2 phosphorylation, LIMK phosphorylation, cofilin phosphorylation, Bad phosphorylation	Regulation of cell motility, Antiapoptotic effect	GRP78	dsRNA-mediated GRP78 knockdown	[76]
In vitro (human proximal tubular epithelial cells, rat renal fibroblasts); in vivo (type 1 diabetic Akita mice)	Primary cells	Protease-α_2_M*	/	FAK phosphorylation, PI3K activation, Akt phosphorylation, TGF-β1 activation, noncanonical YAP/TAZ activation	Tubulointerstitial fibrosis	GRP78	α-GRP78, peptide- mediated GRP78 blockade	[82]
In vitro (rat hippocampal neurons)	Primary cells	MA-α_2_M*	50 nM	NMDA-mediated Ca^2+^ signaling inhibition, NMDAR1 down-regulated	Alteration of neuronal function	LRP1	RAP	[84]
In vitro (human astrocytoma cells)	3121N1	MA-α_2_M*	0.1 μM/0.2 μM/0.5 μM	Wnt/β-catenin signaling antagonism; E-cadherin and N-cadherin upregulation	Tumor suppression	LRP1	α-LRP1, RAP	[81]
In vitro (murine macrophage cells)	Raw264.7	MA-α_2_M*	60 nM	PKC-dependent signaling activation; FAK phosphorylation, β1-integrin endocytic cycling activation	Induction of mesenchymal cellular migration, Cytoskeletal remodeling	LRP1	RAP	[77]
In vitro (murine peritoneal macrophages)	Primary cells	MA-α_2_M*	100 pM	Gq-PLCβ, upregulation of Ca^2+^/DAG, PKC/MAPKs activation, cPLA_2_ phosphorylation, NF-κB activation, CREB phosphorylation	Mitogenesis, Cell proliferation	Unidentified	/	[68]
In vitro (murine peritoneal macrophages)	Primary cells	MA-α_2_M*	100 pM	IP3/Ca^2+^-dependent signaling cAMP-dependent signaling	Cellular proliferation	Unidentified	/	[69]

## Data Availability

No new data were created or analyzed in this study. Data sharing is not applicable to this article.

## Abbreviations

The following abbreviations are used in this manuscript:

α_2_M	Alpha-2-macroglobulin
α_2_M*	Activated alpha-2-macroglobulin
A*β*	Amyloid-beta
Akt	Protein kinase B
APP	Amyloid precursor protein
BRD	Bait region domain
cAMP	Cyclic adenosine monophosphate
CD	Circular dichroism
cPLA_2_	Cytosolic phospholipase A2
CREB	cAMP responsive element binding protein
Cryo-EM	Cryo-electron microscopy
CTGF	Connective tissue growth factor
CUB	C1r/C1s-Uegf-Bmp1
DSC	Differential scanning calorimetry
ER	Endoplasmic reticulum
ERK1/2	Extracellular signal-regulated kinase 1/2
FASN	Fatty acid synthase
4EBP1	Eukaryotic translation initiation factor 4E-binding protein 1
GFAP	Glial fibrillary acidic protein
GPCR	G protein-coupled receptor
GRP78	Glucose-regulated protein 78
JAK	Janus kinase
JNK	c-Jun N-terminal kinase
LIMK	LIM domain kinase
LRP1	Low-density lipoprotein receptor-related protein 1
MA	Methylamine
MAPK	Mitogen-activated protein kinase
MG1–7	Macroglobulin-like domains
MMP-9	Matrix metalloproteinase 9
MTJ-1	Murine tumor cell DnaJ-like protein 1
MT1-MMP	Membrane-type 1 matrix metalloproteinase
mTOR	Mammalian target of rapamycin
mTORC1/2	Mechanistic target of rapamycin complex 1/2
MYC	MYC proto-oncogene, bHLH transcription factor
NCK	non-catalytic region of tyrosine kinase adaptor protein
NF-κB	Nuclear factor kappa B
NMDAR	N-methyl-D-aspartate receptor
PAK	p21-activated kinase
PDK1	3-phosphoinositide-dependent protein kinase-1
PI3K	Phosphoinositide 3-kinase
PKC	Protein kinase C
PLC	Phospholipase C
PLK1	Polo-like kinase 1
PS1	Presenilin 1
p38	p38 mitogen-activated protein kinase
Rab	Ras-related in brain
Rac	Ras-related C3 botulinum toxin substrate
RBD	Receptor-binding domain
SREBP	Sterol regulatory element-binding protein
S6K	Ribosomal protein S6 kinase
STAT	Signal transducer and activator of transcription
TAZ	Transcriptional coactivator with PDZ-binding motif
TED	Thioester domain
TGF-*β*1	Transforming growth factor beta 1
T2D-DKD urine	Type 2 diabetes with diabetic kidney disease urine
UPR	Unfolded protein response
YAP	Yes-associated protein 1
